# Mobile health application for Thai women: investigation and model

**DOI:** 10.1186/s12911-022-01944-0

**Published:** 2022-07-30

**Authors:** Chalermpon Kongjit, Acrapol Nimmolrat, Achara Khamaksorn

**Affiliations:** 1 College of Arts, Media and Technology, Chiang Mai University, Chiang Mai, 50200 Thailand; 2 Research Group of Embedded Systems and Mobile Application in Health Science, College of Arts, Media and Technology, Chiang Mai University, Chiang Mai, 50200 Thailand

**Keywords:** User-centered design, Graphical user interface, Usability, Application functionality, Women’s health application

## Abstract

**Background:**

Women’s mobile health (m-health) applications are currently widely used for health education, medication, prevention of illness, etcetera. However, women are extremely sensitive to their design. While the number of m-health applications for women is increasing, many are of poor quality and have development issues.

**Objective:**

This paper aims to develop and evaluate an m-health application for Thai women based on a user-centred design (UCD). Current women’s m-health applications were investigated to identify any lack of development in usability, functionality and graphical user interface. The results were evaluated and used to create criteria for the trial of a prototype application.

**Methods:**

UCD methodology was used to design a graphical user interface, analyse the application’s functionality, and enhance its usability. Data from thirty female end-users were collected and maintained locally, and thirteen information technology (IT) experts provided feedback on the prototype trial. Interviews and questionnaires were used to gather user data and identify problems.

**Results:**

The average scores of the evaluation by the end-users (n = 30) and IT experts (n = 13) were compared using a t-test statistical analysis. For the first version, the end-users gave higher usability scores (average = 4.440), with no statistical significance and a *P* value of 0.05. In comparison, lower scores for functionality were given by the IT experts (average = 4.034), with no statistical significance and a *P* value of 0.05. For the second version, the average scores from the end-users were higher than those from the IT experts. The highest score was related to usability (average = 4.494), with no statistical significance and a *P* value of 0.05. The lowest score was for the user interface from the group of IT experts (average = 4.084), with no statistical significance and a *P* value of 0.05.

**Conclusion:**

A UCD was utilised to construct a process taxonomy to understand, analyse, design and develop an application suitable for Thai women. It was found from an evaluation of the currently-available women’s m-health applications that usability is their main weakness; therefore, this aspect needed to be prioritised in the new design. According to the results, IT experts’ perspective of the development of an m-health application was different from that of end-users. Hence, it was evident that both end-users and IT experts needed to be involved in helping developers to analyse, prioritise and establish a strategy for developing an m-health application, particularly one for women’s health. This would give researchers an in-depth understanding of the end-users’ expectations.

## Introduction

Mobile health (m-health) applications (apps) are a significant component of daily life in this digital age and provide users with several benefits. These apps are popular tools that can change people’s health behaviour and improve their quality of life [1]. They are widely used for prevention, medication, rehabilitation, etc. [2–9]. They also impact healthcare services in hospitals, care centres and emergency services [10–12]. Based on a study of m-health developer economics, more than 325,000 m-health apps were available in major app stores (e.g. Android and iPhone Operating System (iOS)) at the end of 2017, with more than 3.7 billion downloads (a number which continues to increase daily).

M-health smart technology supports and improves healthcare and medical services in many countries, such as the United States (US), the United Kingdom (UK), Germany, Israel, and Canada [13]. For example, m-health apps were the primary source of health information for almost 28% of Americans in 2017 [14]. Moreover, several researchers have found that women are more likely than men to use digital technologies for online health and medical information [15, 16]. Likewise, more women than men use health apps, according to studies undertaken in the US [17] and Hong Kong [18].

Most of the Thai population regularly uses smartphones, and the number of users is still growing rapidly [19]. Alongside this growth, according to 2018 public health statistics in Thailand, women’s health problems are increasing, especially obesity, diabetes, metabolic diseases, and osteoporosis [20]. There is also an increase in female-related conditions, such as breast cancer and cervical cancer, and a need for careful monitoring throughout pregnancy, childbirth, and the puerperium. In this context, an app based on smartphone technology could help women stay healthy while improving their awareness of diseases to prevent or control them. Several developers are attempting to capitalise on this trend by designing m-health apps for the Thai healthcare system. For instance, the Royal Thai Government has implemented smart technology as a medical service component for mothers using apps such as KhunLook, KidDiary, and MyYaAndYou [21]. Thus, developing women’s m-health applications has huge market potential in Thailand since there are currently insufficient apps to accommodate the large number of users [22].

Health app developers face numerous challenges, and it has been reported that many readily-available low-cost apps were not developed based on research [23, 24]. Therefore, several of them are impractical or unusable due to the limited screen size of m-health devices [25, 26], leading to many uninstallations. Usability remains one of the most important attributes when determining the overall quality of a software system [27]. Still, many apps’ low-quality designs or functional features are often ignored [28].

Despite the benefits of these applications, users are still dissatisfied with them. According to the preliminary results of our trial of the m-health apps in the market (Play Store), the graphical user interface of health applications lacks developmental design. There is too much content, too many unplanned and unnecessary functions, and confusing screen elements. This poor design causes misunderstandings and makes apps uncomfortable and difficult to use.

The variable standard of users’ Information Technology (IT) literacy skills is another issue that needs to be considered when applying smart technology [29, 30]. It is not easy to develop an application that satisfies all women. For instance, inappropriate colours, textures, interactivity and sensitivity may fail to increase their emotional satisfaction, and they will be reluctant to use an app that lacks useful functions. However, a user-friendly app with enough appropriate features will increase women’s motivation to use it [31, 32].

Moreover, different cultural groups have different ways of observing and processing information. For example, the design of Asian user interfaces involves colourful cartoonish and animation elements, while European design presents information in a straightforward and organised manner [30]. Evidence indicates the significance of cultural features in the design of m-health apps, including providing reliable and valid culturally-based design features to encourage users to adopt the m-health User Interface (UI) [33].

This research analyses the available m-health applications for women and designs a new application based on understanding and resolving the problems mentioned above. Investigation of the issues by end-users and IT experts is expected to reveal the reasons for any app design flaws and facilitate the design and development of a new app. It was hypothesised that the two groups’ feedback would not differ significantly.

Therefore, the output of this study consists of a design framework for improving the app UI, usability and functionality. The dual perspective of end-users and IT experts can be used as a guide for developers to analyse, prioritise and establish a strategy for developing an m-health application specifically for Thai women.

### Study objective

This study aims to apply user-centred design (UCD) methodology to research, create and refine the framework for a new m-health app for women. Therefore, the research questions are present as follows:

1. What are the problems with current women’s m-health apps?
2. What is the end-users’ perspective of women’s health apps, and what features do they prioritise?
3. What are the IT experts’ perspectives, and what features do they prioritise?
4. Do end-users’ and IT experts’ opinions differ based on the app prototype evaluation?

## Literature review

This research avoids traditional software engineering methods by using UCD and Usability Design techniques. As the basic re-design and app refinement methods, UCD and Usability Design are examined in this section, along with other research theories and methods. Usability measures, user experience, and related empirical work are also briefly discussed.

### User-centred design process

A UCD process is a scientific approach focussing on the speedy development of a system production process for resolving problems based on users’ needs and incorporating user-centred activities [32]. Although a traditional software development process is commonly used for design and development, this study applied a user-centred process to design a mobile app. This is because a UCD process is faster but focuses similarly on analysis, design, development and testing.

The concept of a UCD process has been applied to great effect in several fields, such as innovation development [34], IT [35], and healthcare development [36, 37]. It uses an iterative process to challenge assumptions, redefine problems, determine user preferences, and create innovative solutions [35] based on the following 4 phases:

- Phase 1—Defining the specification of use. This requires an empathetic understanding of users and the problems to be overcome.
- Phase 2—Defining the specification of user requirements.
- Phase 3—Proposal of several design solutions to satisfy all requirements and selecting one for prototyping.
- Phase 4—Evaluation of the prototype to determine if it provides the best possible solution to each need identified in the first two phases.

Based on the results, a poor design or incomplete prototype can be returned to the previous stages to be corrected or improved.

### Usability design

Usability Design is the primary consideration when creating the graphical interface in this study based on users’ suggestions and theoretical criteria. Usability Design is defined as the product’s performance in terms of ease of access. According to ISO 9241-11, it is “the extent to which a product can be used by specified users to achieve specified goals with effectiveness, efficiency and satisfaction in a specified context of use” [38]. Based on this standard, Jakob Nielsen’s [39] ten general heuristics for user interface (UI) design were used to identify the information required, such as elements, content, size, etc., as shown in Table 1.

**Table 1:** Nielsen’s ten general heuristics

No.	Suggestions
1	Visibility of system status
2	Match between the system and the real world
3	User control and freedom
4	Consistency and standards
5	Error prevention
6	Recognition rather than recall
7	Flexibility and efficiency of use
8	Aesthetic and minimalist design
9	Help users recognise, diagnose, and recover from errors
10	Help and documentation

In terms of the graphical interface design, an effective application must be practical and user-friendly. Therefore, a universal design must be made to benefit all users. Such a design must be easily accessible and usable without being specialised or needing adaptation [40]. Thus, the concept of a universal Usability Design can be applied to a variety of purposes [41, 42] as follows:

1. *Equitable use* All users should benefit from the design, regardless of age, status and ability.
2. *Flexibility of use* The design should accommodate a wide range of individual preferences and abilities.
3. *Simple and intuitive use* The design should be easy to understand, irrespective of users’ experience, knowledge and linguistic skills.
4. *Perceptible information* There should be sufficient information for users to make the most efficient use of the system.
5. *Tolerance for error* The design should minimise errors and the negative consequences of unexpected or unintended actions.
6. *Low physical effort* The design should be efficient and comfortable for users, with minimal repetitive actions or unusual bodily positions.
7. *Size and space for approach and use* All users should be able to approach, reach, manipulate and use the design, regardless of their body size, posture or mobility.

Based on the concepts presented above, it can be seen that Usability Design enhances all users’ levels of access to the system.

### Measure of usability

The users’ ability to utilise the UI must be assessed to ensure that the system designed in this research has a high level of usability. Therefore, user testing determines the UI’s success and the design’s usability [43, 44]. A successful UI must be easy to understand and use. According to published sources, the measure of usability can be divided into the following two categories [42].

### Usability inspection

A heuristic evaluation is useful for inspecting a design interface because it can identify usability problems based on heuristic criteria [45]. Hence, a heuristic evaluation was utilised in this study to inspect the design interface and identify any usability issues identified by the experts and end-users.

### Usability testing

Usability testing involves evaluating the entire development process from the prototype to the completed product based on observing target users’ interaction with the app and obtaining their feedback. This evaluation will result in an application that is efficient, effective and enjoyable to use. Nielsen’s [39] ten general principles were applied to the heuristic evaluation in this research [46].

### Design for women

The design particularities of m-health apps, especially those for women, have no specific theory or framework. A women-centred design has been established to guide designers or researchers in understanding women’s preferences and needs. This concept is similar to a Usability Design, but some characteristics, such as understanding women’s motivation, physiology, experience and perception, are different. Based on a summary of empirical studies [47], Table 2 lists the points and solutions applied in this study to facilitate a better understanding of a women-centred design.

**Table 2:** Suggested Points for a women-centred design

Points	Solution
Motivation	Use plenty of colour, beautiful textures and clear designs
	Apply useful and necessary functions based on a clear understanding of their use
Physiology	Support touching for screen viewpoints, and size and positioning of buttons and text boxes
Perception	Use more organic, amorphous shapes. Display data in wide, spread-out areas with appropriate representation and visibility
Language	Informal. Use symbols or signs

### Gendered dimensions for the use of m-health apps

Sex and gender differences in digital literacy [48], use [49], preferences [50], and the seeking of online health information [15, 16] have been reported in previous digital health technology studies. However, gendered dimensions have received little attention regarding the application of gender-sensitive design. According to some, many of these technologies have been designed for universal users, but not everyone is the same [31].

Previous investigations have shown that women are more concerned about their health and tend to follow a healthier dietary pattern than men [51], and those with a better educational background are more likely to use m-health apps than men with a similar background [49]. This implies that developing trials in digital health technologies from a gender perspective has significant relevance, especially in m-health apps.

Furthermore, few of the many commercially available m-health apps for women have been subjected to rigorous development and evaluation. Derbyshire and Dancey [52] analysed the star ratings and comments from reviews of women’s health apps. Most comments indicated the need for improvement in graphics quality, speed of downloading graphs, device compatibility, and more reputable sources [52]. Also, women complained about the excessive time it took to manually input data, poorly designed apps that crashed or failed to work properly and sync with other devices, and the lack of accuracy [31]. This demonstrates the significance of considering women’s needs and preferences when designing a women-centred m-health app.

### Related empirical studies

The m-health applications presented in this section were based on a scientific approach to developing and evaluating behavioural health interventions. These studies represent the various stages of the development of m-health apps.

Njie-Carr et al. [53] developed an m-health app for older women infected with human immunodeficiency virus (HIV). The process they used to obtain initial feedback on this theoretically-based m-health app consisted of an iterative approach in two phases in which they gathered input from focus groups incorporated with a community advisory board [50].

Teitelman et al. [54] described the development of an m-health app to promote the completion of the human papillomavirus vaccine (HPV) and provide information about how to prevent cervical cancer in young adult women living in economically-disadvantaged urban communities. This app included information, motivational content, a discussion forum, and vaccine completion reminders. The community and provider advisory boards gave feedback on the design at various stages of the app development process [54].

Carrilho et al. [55] explained how a UCD and both qualitative and quantitative approaches were utilised to design and develop an in-app birth plan prototype to establish communication between pregnant women and a healthcare team. Users’ perceptions and experiences of the app were derived from structured and open-ended questions. The app was subsequently improved as the participants completed the tasks and shared their feedback [55].

## Methods

### Experimental sample

The participants in this study were divided into two groups: general target users and IT experts. Random sampling was used to recruit 30 Thai females aged between 18 and 43. Purposive sampling was used to recruit thirteen IT experts with more than 2 years of experience in Chiang Mai Province.

Please note that it is assumed that the age ranges chosen in this study represent women of various generations, including young adults (18–22), adults (23–27), working-age adults (28–32 and 33–37) and older (38 and up).

Note that this research focuses only on problems related to usability and development, not medical benefits. However, a health response team of qualified doctors carried out the research.

The study participants were purposely separated into five age ranges, as shown in Table 3. The respondents’ IT literacy skills were self-determined. Their ability depended on the duration they had been using smartphones. Due to the limited number of participants, not all had a medical need for m-health technology. The findings indicated limitations in the mobile development experience of some of the IT experts in this study.

**Table 3:** Demographics of samples (n = 30)

Points	Characteristic	Percentage (n)	
Age ranges	18–22	26	8
	23–27	20	6
	28–32	16	5
	33–37	23	7
	38 up	13	4
IT literacy skill	Beginner smartphone user (experience of using a smartphone < 1 year)	20	6
	Intermediate smartphone user (experience of using a smartphone > 1 year)	66	20
	High level smartphone user (experience of using a smartphone > 2 years)	13	4

### Experimental framework

**Fig. 1:**
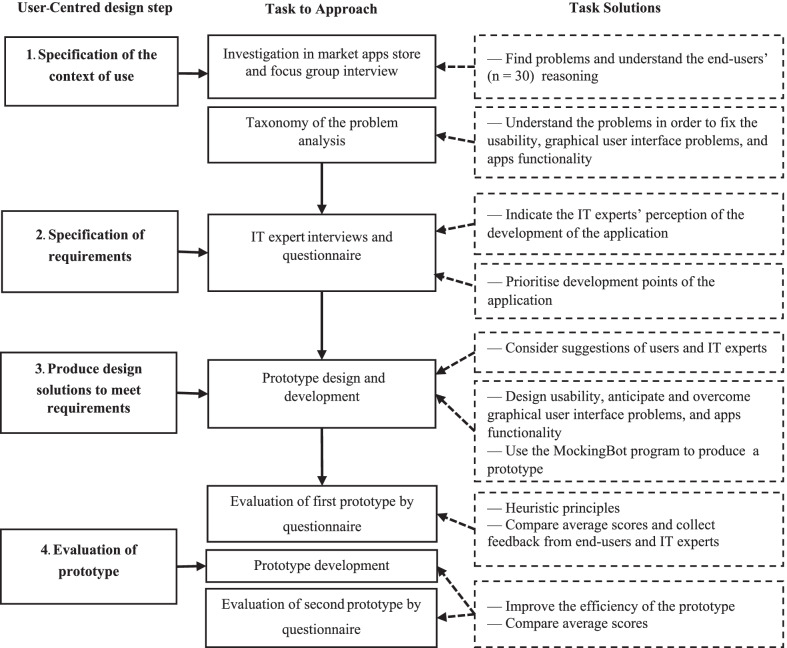
Conceptual framework and research methodology

A series of design processes and methodologies were used to produce an m-health application that could support women’s health, thereby achieving the research objective. A UCD was implemented to identify problems and design appropriate functions, content, and a graphical user interface. The conceptual framework of the research is depicted in Fig. 1. The framework consists of four phases, namely: the specification of the context of use, specification of the requirements, production of design solutions to meet those requirements, and a prototype evaluation.

### Context of use specification

In this phase, the research focuses on a deep understanding of the user needs and the problems related to the usability of existing m-health applications. Keywords were used to classify the existing applications in the Thai language, such as: “women’s period, women’s self-assessment, women and mothers, women’s medicine, women’s personal medical record, women’s weight control, women’s organs, etc.”

Of the 156 apps associated with women’s health supporting the Thai language, only 50 were used in this study. They were selected after considering the following:

- Aim of development (related to women’s health).
- Review scores (more than 4-star rating).
- Application type (the main features of apps associated with women).
- Cost (free or not more than 5 USD).
- Connection type (online request or standalone).

In the questionnaire survey stage, prospective participants were sent an invitation letter (via email). A consent form was also attached to ensure participant permission for their data to be used in this online research. After character analysis, 30 general users were given 10–20 min to test each of the 50 apps over a period of 30 days. Note that devices were not supplied, and connection costs were not supported in this research. Users had to install the apps on their own mobile devices.

A single focus-group was arranged with the general users, and content analysis was used to count the frequency of respondents’ feedback. These users were then randomly separated by name into three groups (10 users per group). Thirty-minute interviews were conducted via Zoom to collect and categorise different problem-related data from each group. General questions for the focus group interviews were:

1. What were your reasons for selecting this application?
2. What points did you consider?
3. What problems did you find when you trialled the application?
4. What points would encourage you to use a particular application?
5. What was your experience with the apps’ interface, function and usability?

This study was conducted solely online. Video recording was not permitted in this research due to ethical reasons. However, four staff took notes of respondents’ answers during the focus group. Note that a health response team consisting of four qualified doctors conducted the entire process.

The next step involved analysing the collected data related to the app’s graphical design and functionality, focusing on two points. The first was the specific problems users encountered when interacting with the apps, and the second was the reason for their positive impression of some of them. The feedback was then analysed and classified to build a taxonomy of the problems and the main reasons for their app choice.

### Specification requirements

In this phase, thirteen IT experts were recruited to share their ideas and provide additional feedback to highlight further problems. They were asked to consider the characteristics of the apps corresponding to those in the previous phase. The characterisation points were summarised in a questionnaire. Each question was designed to determine the core characteristics of a good m-health application and prioritise the reasons for that determination. A numerical scale from one to five was used to quantify qualitative feedback on the design to indicate the IT experts’ perception of the app’s development (Table 7).

### Requirement design solutions

A prototype application was constructed from the findings in the earlier phases of the study and was piloted on Android’s operating system (OS) platform. The prototype contained a list of the contents, features and functions of the graphic user interface and its universal design based on a summary of the experts’ suggestions and the existing taxonomy of the users’ requests. It was developed using the MockingBot program.

### Prototype evaluation

Individual users were sent the link via ‘Line’ (a popular social app in Thailand) to access the first version of the prototype app for installation and evaluation on their devices. They were asked to test it for 30 min and then provide feedback in a questionnaire. Heuristic principles [37, 42] were adapted and applied to test the app’s usability. The evaluation criteria contained in the questionnaire were a modified version of the core usability test criteria of [53] and are shown in Table 8.

In the first round of testing, the scores from the questionnaire were analysed to summarise users’ feedback and a t-test statistical analysis was applied to compare the average scores. The prototype was then refined based on the specific points raised in these findings. This process was repeated for the revised prototype, and again the data derived was analysed. Meanwhile, the IT experts were interviewed to re-check the application’s performance after the modifications (Table 7).

## Results

### Results context of use specification results

The first phase aimed to clearly understand the problems women encountered when using m-health applications and their goal of using them. As mentioned earlier, the initial search yielded 156 applications, 50 of which were selected for a review by general users. The main features of the chosen applications are shown in Table 4. There were 11 categories, which included women’s anatomy, women’s content, comfort, disease and medicine. Applications with these specific characteristics were chosen to acquire an insight into the graphical user interface of existing healthcare apps.

**Table 4:** Main features of apps associated with women in the Google Play store (n = 50)

Type of application	Percentage (n)	
1. Female anatomy	18	9
2. Women’s content	14	7
3. Consolation	12	6
4. Disease and medicine	14	7
5. Blood pressure	12	6
6. Mother	6	3
7. Eyesight and vision	6	3
8. Herbs Medication	6	3
9. Mood assessment	4	2
10.Teeth	4	2
11.Weight control	2	1
12. Hearing	2	1

The frequent use of colour in the apps’ interface was also analysed, and the results are shown in Table 5. Note that colour use is one of the points of consideration for comfort. Colours can be customised to suit the user’s comfort level and directly relate to the user interface. The five* colours most frequently used were white, black, pink, blue and green. Therefore, these colours were incorporated into the prototype’s UI design.

**Table 5:** Frequency of colour used in women’s apps in the Google Play store (n = 50)

Most frequently-used colour	Percentage (n)	
1. White	23	35
2. Black	16	24
3. Pink	16	25
4. Blue	12	18
5. Green	8	13
6. Red	6	10
7. Light blue	5	8
8. Grey	2	4
9. Purple	3	5
10. Brown	2	3
11. etc.	3	5

Most of the issues cited in the general users’ reviews are shown in Table 6, which contains the group feedback, frequency of answers, number of apps and types of problems. The results of the focus group interviews are classified by the most frequent answers (qualitative data). The issues were divided into three types: usability, user interface and functionality. For instance, in 35 of 50 apps, the users initially did not know how to start using the app (24 of 30 found this). Indeed, more user interface issues (6) were found than usability (3) and functionality (3) issues, which indicated that existing healthcare applications tend to lack an acceptable user interface.

**Table 6:** Most frequent answers from focus group questioning (lower end cut off at 10)

Most frequent group answers	Frequency of answers (n = 30)	Number of applications found (average)	Type of problem
User did not know how to start using the app	24*^1^	35*^1^	Usability
Difficult to understand some icons or menus	20*^2^	30*^2^	User interface
Words or explanations were hard to understand	18*^3^	25	User interface
Difficult to read text due to small size	16	26	User interface
Too many unused functions	15	27*^3^	Functionality
Too difficult to understand for use	14	22	Functionality
Many fields had to be input into the application	14	26	User interface
Could not find the ‘return to menu’ button	13	18	User interface
Background colours did not suit the app contents	12	27*^3^	User interface
The application was inappropriate for use	12	13	Usability
App did not fit my size mobile device (impractical)	11	13	Usability
Some functions were unnecessary	10	17	Functionality

*Consideration priority level

**Fig. 2:**
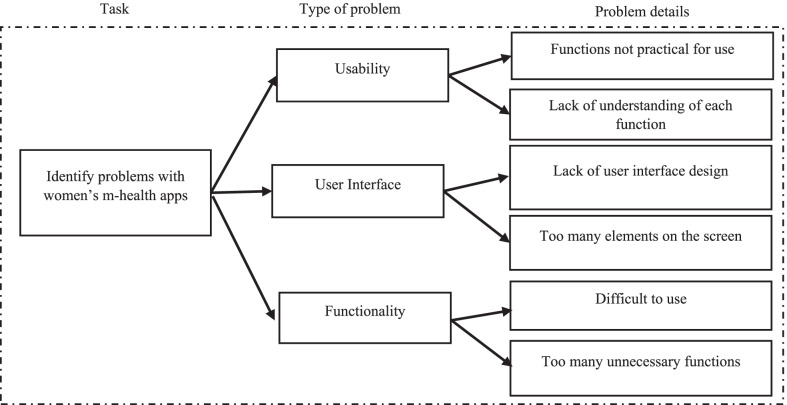
Process taxonomy summary of problems with existing women’s m-health apps

A process taxonomy of each type of problem was constructed based on the above feedback. According to Fig. 2, three aspects of these apps were found to be specifically problematic: usability, UI and functionality. The causes of these problems were identified for later correction in the new app. Examples of user interface-related issues were “Some user interfaces are not suitable for users”, “The user interface design is not compatible with all users”, “I don’t know how to use the application”, “The app’s functions are difficult”, “I don’t know the meaning of the signs or buttons”, “When I clicked, the application didn’t show any results”, and “It was hard to understand some of the functions.”

**Fig. 3:**
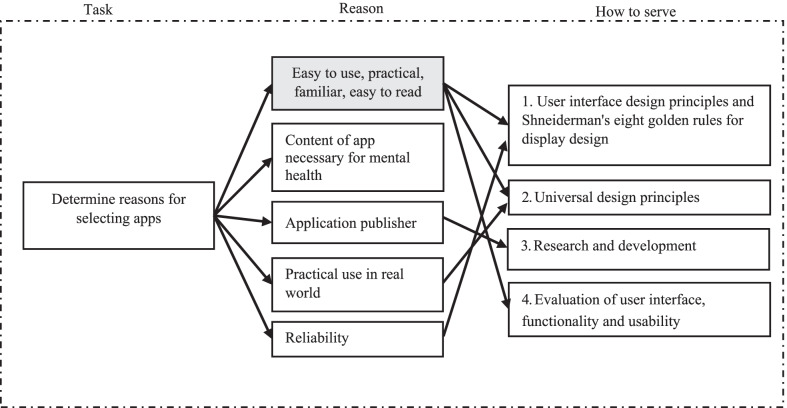
Taxonomy: reasons for selecting applications

To fulfil the research objective, it is necessary to understand why users had a positive impression of some apps and not of others. The reasons for this are shown in Fig. 3 and ranked one to four based on high-to-low frequency. It can be seen that the main reasons users had a positive impression of some apps were that they were easy to use, practical, familiar and easy to read. The taxonomy results were used to identify design points for the prototype app in this study.

### Specification of requirements

The results of the first round of statements designed to collect data from the 13 experts are summarised in Table 7. Points that they considered most significant for developing a health app for women were prioritised.

These qualitative comments helped define the characteristics of the application and were converted into quantitative results using a zero to five rating scale. The statements were based on the classification of m-health apps’ evaluation criteria, focusing on usability, functionality and design (graphical user interface) [54, 55].

The average scores in response to statements 1, 3, 4 and 6 were high (> 4.5) and reasonably homogenous (10 of the 13 experts gave a score of 5). Therefore, from the experts’ perspective, the key points of statements 3, 4, 1 and 6 should be prioritised when considering the characteristics of a women’s ideal health app. This enabled some corrections to be made to the design of the prototype app.

**Fig. 4:**
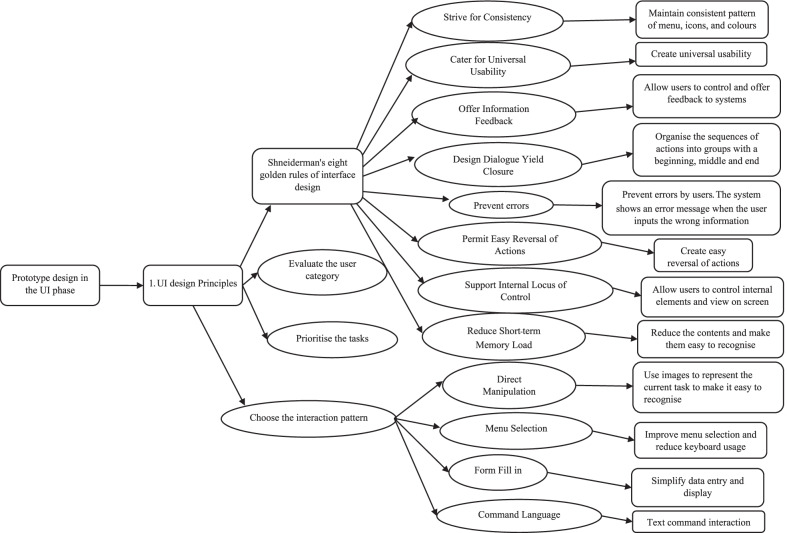
Taxonomy of graphical user interface design for women

The methodology used to design the women’s health application in this study is shown in Table 7. The taxonomy of the graphical user interface design shown in Fig. 4 was based on Nielsen’s [25] ten general principles and Shneiderman et al.’s [56] eight golden rules. It includes (1) the eight golden rules of interface design, (2) evaluation of user categories, (3) prioritisation of tasks, and (4) choice of interaction pattern.

**Table 7:** Summary of IT experts’ comments in round 1

Statements	Experts’ scores					AVG	SD
	5	4	3	2	1		
1. Women’s health applications should be easy to use and customise	10	2	–	1	–	4.615*^3^	0.869
2. Women’s health applications should be designed by understanding users based on their experience	3	10	–	–	–	4	0.438
3. Women’s health applications should contain consistent functions	10	3	–	–	–	4.769*^1^	0.438
4. Women’s health applications should contain necessary functions with practical use	10	2	1	–	–	4.692*^2^	0.630
5. Women’s health applications should contain an image as a universal symbol, readable text size, and icons or menus that make them easier to understand	9	1	2	1	–	4.384	1.043
6. Women’s health applications should contain appropriate colours and elements and clearly visible content spaces	11	1	1	–	–	4.769*^1^	0.599

*Level of priority

**Fig. 5:**
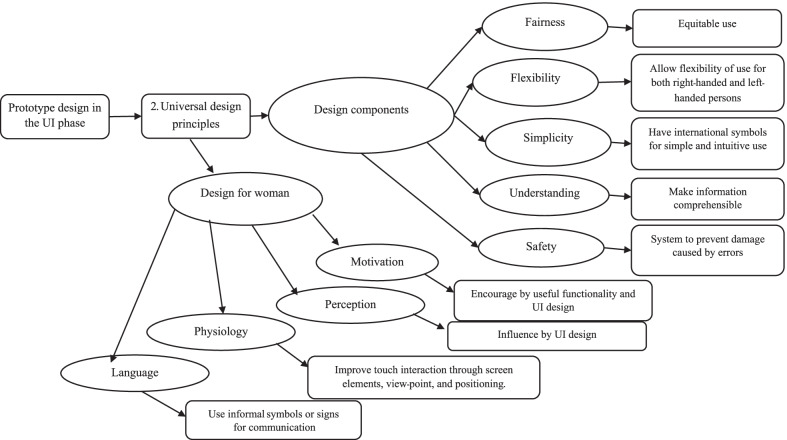
Taxonomy of universal design principles

The taxonomy of the universal design of the graphical user interface illustrated in Fig. 5 was based on the users’ problems and the IT experts’ suggestions. It consists of (1) design for women and (2) the components of a universal design.

This design uses universal design principles for women to increase its usability. Since female users are different from men, using appropriate colours, textures, interaction, and refinement reinforce their emotional satisfaction. Hence, the findings in this phase were used to develop the app prototype with women’s anatomy or women’s content in mind. The frequency of colour use, application problems, design taxonomy, end-users’ and IT experts’ suggestions were considered in the next phase.

### Production of required design solutions

Since the results of the previous phase enabled the developer to understand the required characteristics of the UI, a prototype application with female-related content was developed and written for the Android OS platform using the MockingBot program. The design of this prototype (as shown in Table 8) contained the following five essential functions: a period calendar, self-assessment, personal notes, counselling, and notification reminders.

**Table 8:** Analysis of the feminine functions of the prototype app

Function	Aim
Period calendar	This function is used to calculate upcoming period dates and fertile windows
Self-assessment	This function is used to record body temperature, weight and height. The data is visualised by plotting it on a graph combined with the period calendar
Personal note	This function is used to record features such as period symptoms, daily notes and other information not included in the self-assessment function
Counselling	This function provides a counselling feature that involves chatting with doctors
Reminders and notifications	This function provides reminders and push-notifications for the next period, fertile window and stage of pregnancy. Users can enable and disable notifications for each event

**Fig. 6:**
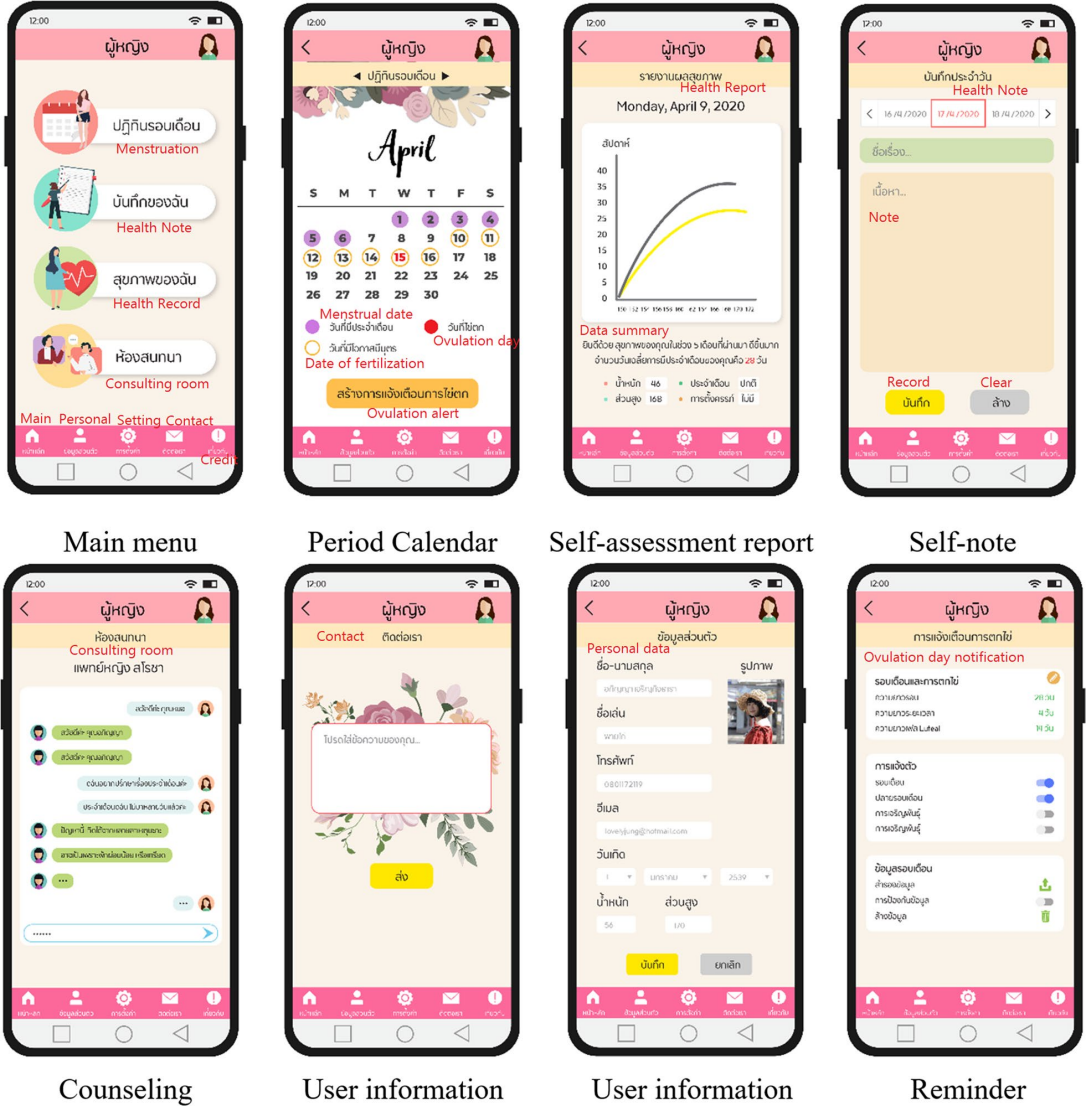
Graphical user interface and usability design for developing the prototype app

Screenshots of each function are shown in Fig. 6, together with the main menu and user information. The five colours most used in the existing apps chosen by Thai women were used for this prototype to attract Thai female users and enhance their acceptance of the new app.

In fact, Thai users’ choice of colours was found to be different from those preferred by foreigners [57]. Based on the frequency of colours in the apps associated with women in the Google Play store (as shown in Table 5), Thai users favoured lighter tones and a mixture of contrasting colours. Therefore, this information was used to establish a strategic colour design throughout the new application.

### Prototype evaluation

**Fig. 7:**
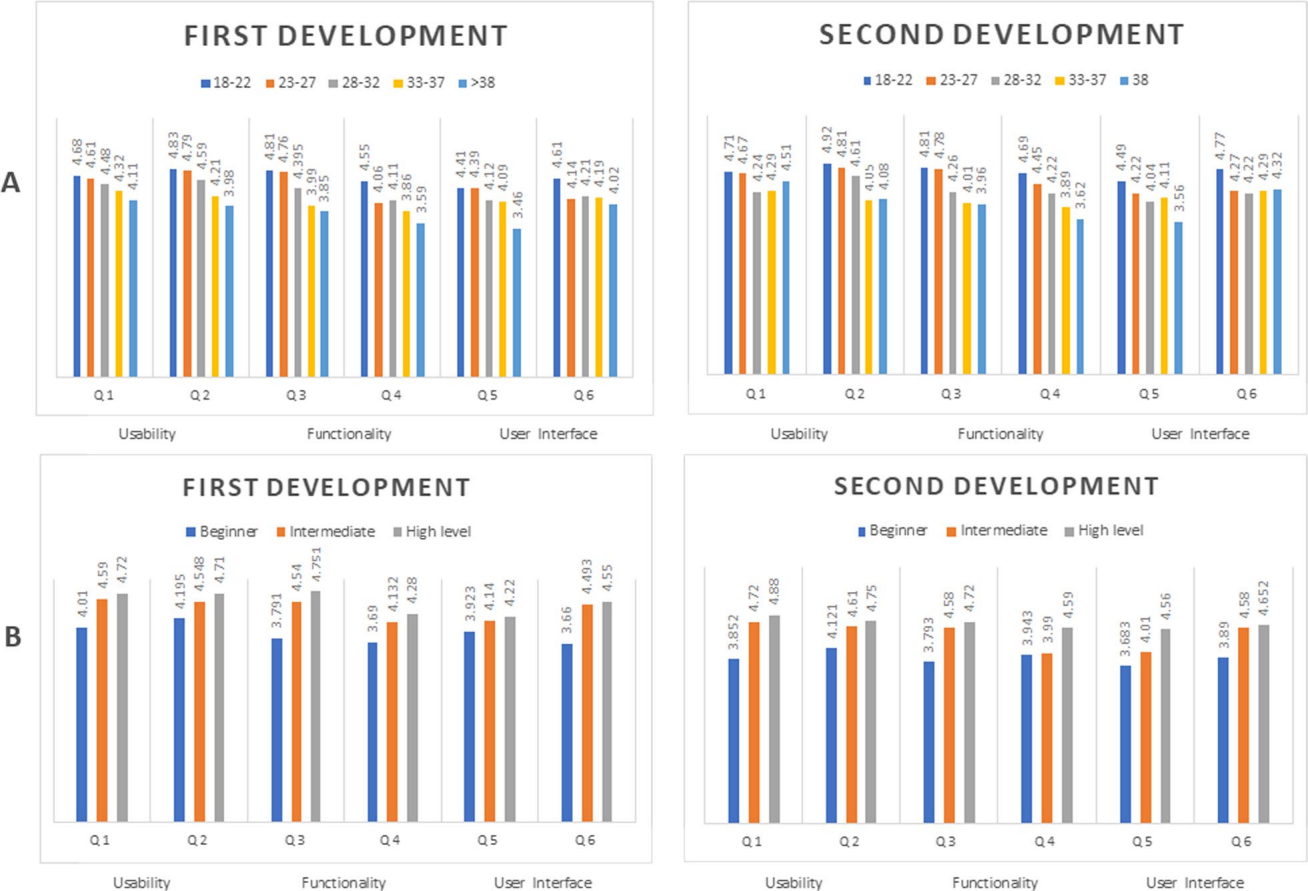
Average scores of each question with end-users separated by age range (**A**) or IT literacy skills (**B**)

**Fig. 8:**
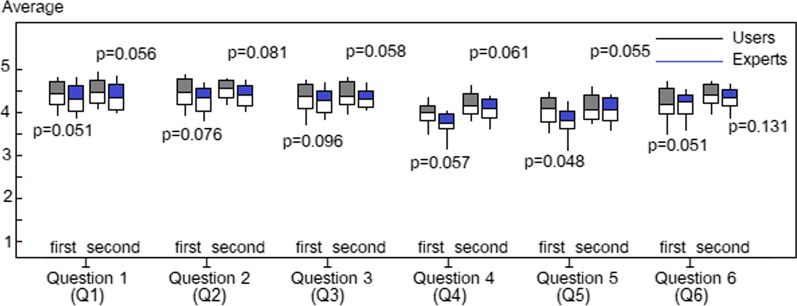
Average scores based on t-test statistical analysis

The first prototype was evaluated through a questionnaire completed by end-users and IT experts. After the prototype was released and pilot-tested, responses were categorised into three dimensions: users’ requirements, application functionality and graphical user interface. The criteria for the evaluation, which were modified from those of [51], are shown in Table 9. The users’ feedback results, separated by age and IT literacy skills, are shown in Fig. 7A, B. The average scores and standard deviation of each question in both groups are presented in Fig. 8. The difference between the groups was determined by the *P* value when *P* < 0.05.

**Table 9:** Evaluation criteria

Questions	Evaluation criteria
*App user’s requirements (usability)*	
Q1	1. Practical and understandable when used
Q2	2. Simple design
*Use of app’s functionality*	
Q3	3. Provides accurate and reliable results
Q4	4. Compatibility between core features and user interface
*Graphical user interface*	
Q5	5. Consistent pattern of menus, icons, colours and fonts
Q6	6. Appropriate screen size and clearly visible

As shown in Fig. 7A, older users gave lower scores than younger ones. The lowest score was given to Q5 regarding the interface elements. Users commented they found it hard to use the functions due to the menu size, unclear icons, inappropriate colours and font size. On the other hand, users with a high level of IT skills gave the app higher scores than beginners (Fig. 7B). The latter were found to be older users. Hence, users’ IT background appears to be a significant factor in assessing the app’s performance, especially for those unfamiliar with m-health applications. The results of each criterion were assessed and compared between the expert and end-user groups, as shown in Fig. 8.

The following observations were made from the first round of the evaluation;

1. Q1 and Q2 were given a high score by both end-users and experts, with sample comments like “I am very new to m-health apps, but this one is easy to navigate”, “It is easy to enter text or numbers”, and “The screen is appealing to women”. These results indicated that the prototype application was perceived to be a design that meets users’ requirements in terms of practical use and simplicity. There was no difference between the two groups, with *P* > 0.05.
2. Q3 was given a high score by both end-users and experts, with sample comments like “The results of the period calculator are accurate”, “I appreciate the self-assessment result of tracking periods”, and “The reminder of an upcoming period is very useful and accurate”. These results indicated that the prototype application is accurate and reliable. There was no difference between the two groups, with *P* > 0.05.
3. Q6 was given a high score by both end-users and experts, with sample comments like “The app’s content on the screen fits my phone” and “The application has a simple and friendly writing style, making it easily readable”. These results indicated that the prototype app’s screen size was appropriate and clearly visible. There was no difference between the two groups, with *P* > 0.05.
4. Q1, Q2, Q3, and Q6 were given higher scores than Q4 and Q5. Points with an average score of nearly 4.0 needed to be improved. These were related to incompatibility between the functions and interface elements, such as the size of menus, size and positioning of icons, colours and font sizes.
5. Both end-users and experts gave Q4 a low score. The experts perceived that the core features were inappropriate for functionality, noting that “The function is not practical for the calculation of users’ information” and “The function has no clear purpose, which confuses users”. It was noted that the average score of all users was *P* = 0.057 the first time and *P* = 0.061 the second time.
6. Both end-users and experts gave Q5 a low score. The experts perceived that the menus’ size and positioning of icons, colours, and font sizes were inconsistent. They commented that the standard button size was too small and suggested increasing it and removing the menu explanation. It was noted that the average score of all users was *P* = 0.048 the first time and *P* = 0.055 the second time.
7. The results of the statistical analysis demonstrated that the only significant difference between the end-users and IT experts was related to Q5 (*P* = 0.048). The end-users liked this pattern more than the experts did. This indicated that the latter perceived that the menus, icons, colours and font size on the graphical user interface of the prototype needed to be improved to enhance users’ experience.

The core features of the UI of the prototype app were improved based on these findings by altering the menu, font and icon sizes, repositioning the icons and changing colours to achieve greater usage efficiency and user acceptance. The revised version also had the menu explanation removed to reduce the screen size. Some of the instant comments were “the menus are too small; increase the size but consider the proportion of the screen”, “The menu explanation reduces the space on the screen. Replace it with signs or symbols”, “increase the task screen”, “increase the font size”, and “Change signs and symbols to make them easier to understand”.

**Fig. 9:**
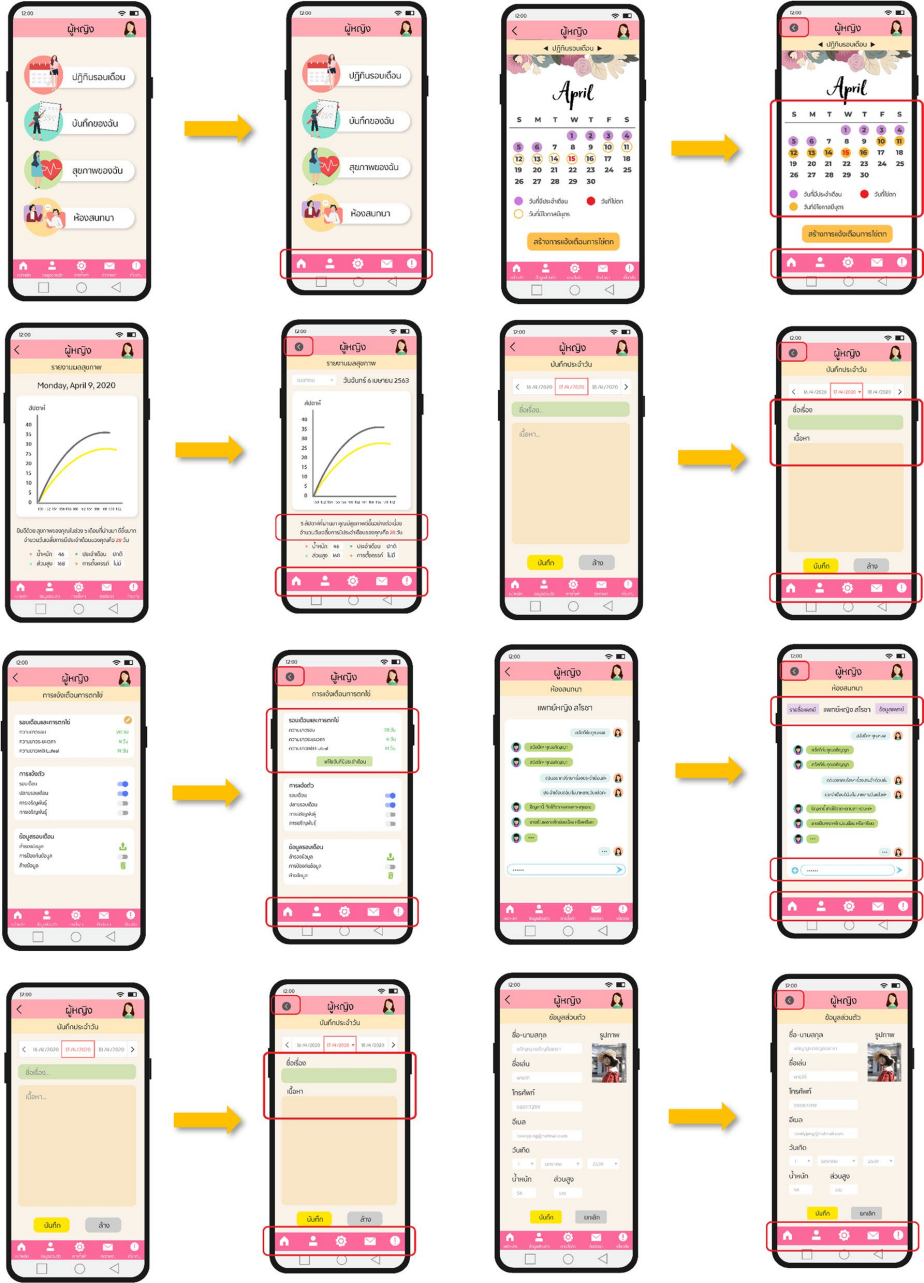
Before and after corrections

The universal symbol for communication was also changed to increase users’ perception, and the majority of the text displayed was fixed by increasing font size. In addition, inadequate command elements, such as interactive buttons, labels and text in textboxes, were corrected to enhance users’ experience. The screenshot results are shown in Fig. 9.

In the second round of evaluation, the lower scores and comparison of the average scores were used to consider which points needed correction in the second prototype.

The average score of Q4 and Q5 was observed to have increased to *P* = 0.061 and *P* = 0.055, respectively, in the second round. This indicated that these improvements had met the criteria of the corrected points, and the trend of the users’ feedback scores was similar. Interestingly, the average Q1, Q2, Q3, and Q6 scores also increased, indicating that improving the app’s functionality had also increased users’ satisfaction. There was no difference between the two groups, with a *P* value of 0.05.

The functionality and graphical user interface were the factors that had most affected users’ satisfaction. Some of the sample comments were, “I prefer the new main buttons more than the previous version because they are more visible and they look nice”, “The counselling channel and graphical elements are more helpful with the addition of the list of doctors and their profiles”, “The chat box can be better operated with fingers”, and “I can edit my information better”. These comments indicated that the revised version had increased the efficiency of the app’s usage and users’ satisfaction.

Furthermore, since the comparison did not indicate a significant difference between end-users and IT experts, the new version appeared to meet the needs of both groups.

The evaluation was based on Lewis’s evaluation criteria with a focus on users’ requirements, the application’s functionality and the graphical user interface.

The results of the second round evaluation by the 13 IT experts were assessed in an interview based on a modified version of the questions in Table 7 to determine whether or not the application met their expectations. As can be seen from Table 10, the majority of their primary concerns had been addressed.

**Table 10:** Summary of results of experts’ evaluation in the second round

Questions	Expert scores rating					AVG	SD
	5	4	3	2	1		
1. This women’s health application provides high usability, being easy to use and customise. Do IT experts agree with this point?	11	2	0	–	–	4.846*^1^	0.376
2. This women’s health application is clearly understandable based on users’ experience. Do IT experts agree with this point?	10	3	0	–	–	4.769*^2^	0.439
3. This women’s health application contains consistent functions. Do IT experts agree with this point?	7	6	0	–	–	4.538*^3^	0.519
4. This women’s health application provides the necessary functions and is practical to use. Do IT experts agree with this point?	4	9	0	–	–	4.308	0.480
5. The universal symbol image, size of text, and icons or menus make it easier to understand this women’s health application. Do IT experts agree with this point?	7	4	2	–	–	4.385	0.768
6. This women’s health application uses appropriate colours and elements, and content spaces are clearly visible. Do IT experts agree with this point?	10	3	0	–	–	4.769*^2^	0.439

*Primary concern rating

## Discussion

Although many m-health applications have been developed for commercial purposes, few were developed based on evidential research or research studies. Most of the existing apps were found to have unnecessary functions and inappropriate graphical user interfaces that annoy users and reduce their usability [26, 27, 58, 59].

An iterative process of continuous testing and refining has been found to solve many problems in the healthcare realm. Yet, many of these m-health applications have not undergone repeated testing to ensure they meet their objective. This includes m-health apps that, despite being purported to be for women, are ignorant of individual users’ needs and lack user customisation and functional tasks and content.

According to empirical literature [26, 27], user interface, functions, and usability are the main factors that still challenge designers and developers of m-health applications. Although they are commonly used worldwide, most high-performance health apps are based on the English language, making them useless for non-English speakers or those with a low level of IT literacy. Hence, there is a need to research and develop local m-health apps for women in their own language.

Therefore, to fit the context of Thai healthcare, a modified design framework was produced in this research for developing an app to solve problems related to the user interface, functionality and usability. The design framework of the app was based on a UCD process [29], an appropriate development methodology previously used to develop several m-health applications [32, 60].

An iterative process with a user-centred design was utilised in this study to tackle the critical challenges before presenting a final prototype app to users for testing. In addition, the results of interviewing IT experts indicated that the most significant features of the app were usability, functionality and UI design [55]. Functionality was evidently more important than other aspects from an IT perspective. Therefore, close attention was paid to functionality design, usability and UI (in that order of priority) when developing the new app [27, 56].

However, it was found from the results of the focus group interview that usability was the main issue for end-users, rather than the UI and functionality. According to user feedback in similar investigations, the most significant problematic features of currently women’s m-health apps are the frequency of colour use, interface elements and functionality [57, 59]. Asian users enjoy an abundantly colourful user interface with cartoonish animation elements, while Europeans prefer information to be presented in an organised clinical way [30, 31].

The women’s m-health application prototype was designed and analysed based on taxonomies of UI design and universal design principles. An analysis of the app’s essential functions was made from the perspective of both end-users and IT experts by comparing the average score of a t-test statistical analysis of the two groups. In the first round of the evaluation related to usability, there was no difference between the two groups’ average functionality scores, with a *P* value of 0.05. In contrast, the two groups’ average scores for UI design were different in terms of the consistent pattern of menus, icons, colours and fonts, with a *P *value of 0.048. Therefore, the perspectives of the two groups were inconsistent.

Several researchers [61–64] mention that UCD frameworks have been developed to create m-health apps because the involvement of end-users in the development process is beneficial for a clear understanding of their needs. Hence, a revised prototype app was produced with improvements based on feedback from the first round of testing.

Feedback from the second testing round indicated that end-users and IT experts were satisfied with the corrections in the revised version. The inconsistent perspectives of the two groups had been rectified, and their average score was similar, with a *P* value of 0.05. Therefore, the two groups’ evaluation of the prototype was the same, indicating the research hypothesis was accepted.

Since the graphical user interface and usability were found to have a more negative effect on most users than the app’s functionality, this process facilitated the creation of an effective m-health application that responds to women’s needs.

The last result of the IT experts’ evaluation in the second round of testing indicated that the most significant factor had changed due to reacting to the end-users’ problems and lack of design [22, 23]. The IT experts agreed that usability must be prioritised when developing the app, and the user interface and functionality design should be the second and third priorities, respectively [20, 21, 23, 24].

Therefore, by focussing on the application’s functional performance rather than other points, the IT experts miscalculated the design priority in this study due to their different perspective. As a result, the app’s functions might not have performed well for end-users, especially Thai women with low levels of IT literacy. As mentioned earlier, the use of the system is directly related to end-users’ IT skills. An application is unlikely to be popular with older groups if it is highly functional but ignores the significance of clear usability. Hence, Usability Design is the first factor that needs to be considered, together with the user interface and functionality, when designing an application for older women.

Lastly, many researchers [57, 65, 66] who have studied the influence of colour over the years have found that men and women prefer significantly different colours. Therefore, it is necessary to use a strategic colour design when developing an m-health application for Thai women. The women surveyed in this study favoured colours such as pink, which is strongly associated with youthful femininity and white, which is related to purity and innocence [67, 68]. However, there was an interesting difference between the choice of colours of Thai users and foreigners. Based on the frequency of colour use, lighter tones and a mixture of colours between dark and light tones are generally favoured by women [69]. Curiously, black is preferred to pink, blue, and green in applications for Thai women, despite not being a universally-loved colour by most females. This indicates that app colour customisation can be used to put users at ease.

It was also found that some designs are unsuitable for Thai users. For instance, while most users could easily identify the exit labels in the first version of the prototype, Thai users did not understand the icons. Therefore, functionality is not likely to encourage Thai users to use an app they consider difficult to use.

## Conclusion

An examination of the m-health apps for women currently available for Thai women confirmed the need to design and develop a more efficient application. The research questions were answered, as shown below.

Firstly, three main problems related to the usability, UI and functionality of current m-health apps were highlighted in a trial by Thai women. It was also found that functionality alone cannot encourage Thai women to use an app they consider awkward to use.

Secondly, end-users with different levels of IT literacy confirmed that usability, rather than the perfect amount of functions, was the most significant element encouraging them to use a women’s m-health application. The user interface was the second most significant element.

Thirdly, it was found that IT experts’ perspective of developing a women’s m-health application was different to that of end-users. The application’s functionality was the most significant factor for IT experts, rather than the usability and UI.

Lastly, it was evident from the research that the joint involvement of end-users and IT experts is essential to enable developers to analyse, prioritise and establish an effective strategy for developing an m-health app for Thai women. Obviously, there is no perfect way to design such an application. However, the application designed and developed in this research is based on a systematic method and conceptual framework and illustrates that an efficient m-health application for Thai women is possible. By using a strategy based on User-Centred Design, extracting knowledge from IT experts and consulting end-users to determine problems, it *can* be done.

### Limitations

Four limitations are notable in this research. First, the study sample was restricted to a single group of end-users (educated women who use smartphones and live in Chiang Mai); this limited the number of end-users and IT experts available.

Second, due to the limited number of participants mentioned above, it could be said that the test evaluation criteria were modified to recruit sufficient research participants (e.g. The number of questions was reduced). This leads to the conclusion that the number of participants is insufficient to represent all Thai women.

Thirdly, due to the specific design of this prototype for basic menstrual tracking, there are limitations in its use by pre-menopausal adolescents and post-menopausal adults. Therefore, future research could develop and analyse a health application for older women.

Lastly, two iterations to improve the application design are insufficient to revise the application prototype adequately. A third session of product testing and revision would be advantageous.

## Data Availability

The datasets generated and analysed during the current study are not publicly available due to privacy and/or ethical restrictions but may be available from the corresponding author on reasonable request.

## Abbreviations

App: Application
HIV: Human immunodeficiency virus
HPV: Human papillomavirus
iOS: IPhone operating system
IT: Information technology
m-health: Mobile health
OS: Operating system
UCD: User-centred design
UI: User Interface
UK: United Kingdom
US: United States
USD: United States Dollar
